# „In Bewegung“

**DOI:** 10.1007/s43594-022-00067-8

**Published:** 2022-10-11

**Authors:** Stefan Raid, Nils Neuber, Peter Lautenbach

**Affiliations:** 1 Deutsche Sportjugend, Hamburg, Deutschland; 2 Westfälische Wilhelms-Universität Münster, Münster, Deutschland; 3 Deutsche Sportjugend, Frankfurt am Main, Deutschland

## Liebe Leserinnen und Leser,

es ist etwas in Bewegung geraten: Im Bewusstsein um die enormen Einschränkungen für Kinder und Jugendliche im Zuge der Covid-19-Pandemie und speziell um den daraus resultierenden Bewegungsmangel haben Entscheidungsträger*innen reagiert. Der nach einem Appell der Deutschen Sportjugend (dsj) und des Deutschen Olympischen Sportbunds (DOSB) von der Bundesregierung in Angriff genommene „Bewegungsgipfel“ gibt nun den Weg vor: Handeln ist das Gebot der Stunde, und nur wenn Bewegung Chef*innen-Sache ist und als Querschnittsaufgabe in allen Ressorts gedacht wird, können die Rahmenbedingungen für ein gesundheitsorientiertes und bewegtes Aufwachsen von Kindern und Jugendlichen geschaffen werden. Die Tür steht jetzt weit auf, um im Bereich des Kinder- und Jugendsports entscheidend weiterzukommen.

Aufwachsen braucht Bewegung. Und beides zusammen benötigt verlässliche, aktuelle Daten. Den Weg zu mehr Berücksichtigung des Kinder- und Jugendsports in der Forschungslandschaft sprechen *Nils Neuber* und *Ulrike Burrmann* in ihrer Einführung zum 2022 erschienenen Sammelband „Kinder- und Jugendsportforschung in Deutschland – Bilanz und Perspektive“ an (er ist in dieser Ausgabe von *Forum Kinder- und Jugendsport* rezensiert). Die Wissenschaftler*innen stellen dabei ernüchtert fest: „Die letzte große Längsschnitterhebung zu Wirkungen des Jugendsports liegt 20 Jahre zurück.“ Es ist also höchste Zeit für ein bundesweit repräsentatives Kinder- und Jugendsport-Panel, zumal alle aktuellen Umbrüche des Kindes- und Jugendalters gleichermaßen den Kinder- und Jugendsport betreffen – ob das ein verändertes Sporttreiben und Rezeptionsverhalten in der Pandemie ist, die Diversifizierung von Sportsettings und -angeboten, der Ausbau von Ganztagsschulen mit dem geringeren Anteil an gestaltbarer Freizeit als Folge, die Mitgliederverluste in Sportvereinen während der Pandemie vor allem bei Kindern und Jugendlichen oder die Verschärfung sozialer Ungleichheiten auch im Sport und nicht zuletzt die Frage, wie sich freiwilliges und ehrenamtliches Engagement in diesem Kontext entwickelt hat.

Ein entsprechendes Forschungsgroßprojekt wird inzwischen von vielen Seiten gefordert, allerdings müssen die verschiedenen Ansätze, Motive und Interessen abgestimmt werden, um in eine gemeinsame Richtung zu stoßen. Das Zeitfenster ist günstig, das Thema auf der politischen Agenda gelandet. Selbstverständlich sollte das Forschungsprojekt inhaltlich und methodisch sorgfältig überlegt sein. Der Forschungsverbund der Deutschen Sportjugend und der Forschungsverbund Kinder- und Jugendsport NRW haben dazu bereits konkrete Vorschläge vorgelegt. Es müssen aber auch die politische Bereitschaft und die finanziellen Grundlagen gegeben sein, um wirklich Bewegung in die Sache zu bringen.

Denken wir über die so wichtige Förderung der Bewegung von jungen Menschen nach, sollten wir nicht vergessen, dass sie in einem anderen europäischen Land dem Krieg ins Auge schauen. Was bedeutet der Krieg für die ukrainischen Kinder und Jugendlichen? Die momentane Arbeit der ukrainischen Jugendverbände und die Rolle respektive das Potenzial des Sports dabei skizzieren* Natalia Shevchuk* und* Matthias Starz* in ihrem Fachbeitrag „Zwischen Krieg, Flucht und Jugendarbeit“. Wie wirkungsvolle Hilfe gestaltet werden kann, greift auch unsere Rubrik „Das aktuelle Stichwort“ auf. Diesmal haben darin *Behzad Borhani* und *Nina Reip* zu „Flucht“ geschrieben.

Im ersten Forschungsbeitrag dieser Ausgabe hinterfragen *Sebastian Gehrmann, Christine-Irene Kraus, Natalia Fast, Christa Kleindienst-Cachay und Valerie Kastrup *das Sportengagement von Mädchen mit und ohne Migrationsgeschichte. Ihre Ergebnisse zeigen, dass Mädchen mit internationaler Geschichte seltener Sport treiben und vor allem türkeistämmige Mädchen und solche aus Ländern der ehemaligen Sowjetunion seltener partizipieren. Auf der anderen Seite erhöht der Besuch einer Gesamtschule und vor allem eines Gymnasiums die Wahrscheinlichkeit der organisierten Sportausübung deutlich. Auch Sportartpräferenzen haben die Forscher*innen in ihrer spannenden Untersuchung unter die Lupe genommen. Wie bei *Forum Kinder- und Jugendsport* üblich, reagieren Praktiker*innen aus dem Kinder- und Jugendsport mit Dialogen auf den Forschungsbeitrag: *Tim Wierling* von der Deutschen Gewichtheberjugend geht in seinem Kommentar auf die Frage ein, was Vereinssport tun kann, um für Mädchen und junge Frauen attraktiv zu sein.* Inge Voigt-Köhler* und* Marlo Mintel *liefern begleitend einen Projektbericht von einem Mädchen-Sport-Camp im Kanusport in Bremen. Solche Camps motivierten 2021 und 2022 Mädchen und junge Frauen bundesweit und in vielen Sportarten zu sportlichen Aktivitäten.

Um die generellen Bedürfnisse von jungen Menschen zwischen 14 und 29 Jahren und wie darauf reagiert werden kann, geht es im Interview mit Jugendforscher *Simon Schnetzer*. Das Fazit aus seiner Trendstudie „Jugend in Deutschland – Sommer 2022“ lautet: Die Jugend kommt nicht aus dem Dauerkrisen-Modus von Klima, Krieg, Corona heraus und ihre Einstellungen wie auch Bedürfnisse verändern sich stark. In unserem Gespräch hat der Forscher auch einen konkreten Ratschlag für den Vereinssport parat.

Bei Corona hakt in dieser Ausgabe aus Sicht einer Medizinerin und Sportwissenschaftlerin *Christine Joisten* ein. Ihr Fachbeitrag beleuchtet den Bewegungsmangel und mögliche gesundheitliche Auswirkungen der Covid-19-Pandemie auf Kinder und Jugendliche und fragt, was für einen gesunden Lebensstil der Jugend zu tun wäre. Joisten hofft auf verankerte Strukturen zur Förderung gesundheitlicher Chancengleichheit für Kinder und Jugendliche und warnt vor einem „Strohfeuer des Aktionismus“.

In einem weiteren Forschungsbeitrag betrachtet *Benjamin Zander* jugendliche Peergroups und wie das Sporttreiben in der Schule und in der Freizeit von ihnen kollektiv wahrgenommen und bewertet wird – eine Arbeit, die neben Erkenntnissen auch Fragen aufwirft, etwa inwieweit beide Sportbereiche miteinander kompatibel sind. Der kommentierende Dialogbeitrag dazu von *Diana Küster* und *Oliver Kauer-Berk* führt grundsätzlich ein und erläutert, wie sich Schul- und Vereinssport über den Erfahrungsraum Peergroup gemeinsam weiterentwickeln könnten.

Weitere wichtige Themen finden auch in dieser Ausgabe Platz. *Kathrin Kohake* und *Alfred Richartz *thematisieren in ihrem Fachbeitrag die Prävention von Gewalt in pädagogischen Beziehungen sowie das Sicherstellen des Kindeswohls im Kinder- und Jugendsport. *Selina Seemüller, Anne Kerstin Reimers* und *Isabel Marzi* haben in ihrem Forschungsbeitrag Grundschulkinder anhand der Photovoice-Methode zu ihrem Schulweg befragt. Ihre Erkenntnis: Insbesondere die elterliche Erlaubnis, eine mangelnde fahrrad- und fußgängerfreundliche Infrastruktur und rücksichtslose motorisierte Verkehrsteilnehmer*innen hindern Kinder an einem aktiven und eigenständigen Zurücklegen des Schulwegs – was wiederum ein Ansatzpunkt für Interventionen zur Bewegungsförderung sein könnte.

Wie ein Kooperationsprojekt mit der Wissenschaft erfolgreich den offenen Ganztag bewegt, zeigt *Kathrin Aschebrock* in ihrem Fachbeitrag. Dabei erläutert sie gleichzeitig den Einsatz kognitiv förderlicher Spielformen. Last, but not least, und hier haben wir uns mit Bedacht für den Wechsel ins Englische entschieden, stellen *Sergio Lara-Bercial, Gary Hodgson, Julian North und Nicolette Schipper-Van Veldhoven *den ICOACHKIDS-Ansatz und dessen „10 Golden Principles for Coaching Children“ vor. Im Rahmen ihres englischsprachigen Fachbeitrags wird auch erläutert, wie die dsj in dieses nicht gewinnorientierte und international ausgerichtete europäische Gemeinschaftsprojekt eingebunden ist.

Bitte bleiben Sie, bitte bleibt Ihr weiter in Bewegung – zum Wohle der Kinder und Jugendlichen. Dafür wünschen wir Ihnen und Euch viele neue Informationen und Anregungen und viel Freude beim Lesen!

**Stefan Raid**

(für die Institutionellen Herausgeber*innen)

**Nils Neuber**

(für die Herausgeber*innen der Forschungsbeiträge)

**Peter Lautenbach**

(für die Herausgeber*innen der Fachbeiträge)

